# The Curious Case of Earthworms and COVID-19

**DOI:** 10.3390/biology10101043

**Published:** 2021-10-14

**Authors:** Janeck J. Scott-Fordsmand, Monica J. B. Amorim

**Affiliations:** 1 Department of Biosciences, Aarhus University, 8600 Silkeborg, Denmark; 2 Department of Biology & CESAM, University of Aveiro, 3810-193 Aveiro, Portugal; mjamorim@ua.pt

**Keywords:** nanoparticles, viruses, immunity, mechanisms, invertebrate, lysenin

## Abstract

**Simple Summary:**

Earthworms have been used for centuries in traditional medicine, and more than a century ago were praised by Charles Darwin as one of the most important organisms in the history of the world. These worms are well-studied with a wealth of information available, for example on the genome, the gene expression, the immune system, the general biology, and ecology. These worms live in many habitats, and they had to find solutions for severe environmental challenges. The common compost worm, *Eisenia fetida,* has developed a unique mechanism to deal with intruding (nano)materials, bacteria, and viruses. It deals with the intruders by covering these with a defence toxin (lysenin) targeted to kill the intruder. We outline how this mechanism probably can be used as a therapeutic model for human COVID-19 (Severe Acute Respiratory Syndrome Coronavirus 2, SARS-CoV-2) and other corona viruses.

**Abstract:**

Earthworms have been used for centuries in traditional medicine and are used globally as an ecotoxicological standard test species. Studies of the earthworm *Eisenia fetida* have shown that exposure to nanomaterials activates a primary corona-response, which is covering the nanomaterial with native proteins, the same response as to biological invaders such as a virus. We outline that the earthworm *Eisenia fetida* is possibly immune to COVID-19 (Severe Acute Respiratory Syndrome Coronavirus 2, SARS-CoV-2), and we describe the likely mechanisms of highly receptor-specific pore-forming proteins (PFPs). A non-toxic version of this protein is available, and we hypothesize that it is possible to use the earthworm’s PFPs based anti-viral mechanism as a therapeutic model for human SARS-CoV-2 and other corona viruses. The proteins can be used as a drug, for example, delivered with a nanoparticle in a similar way to the current COVID-19 vaccines. Obviously, careful consideration should be given to the potential risk of toxicity elicited by lysenin for in vivo usage. We aim to share this view to activate its exploration by the wider scientific community while promoting a potential therapeutic development.

## 1. Introduction

Earthworms have been used for centuries in traditional medicine [1], and more than a century ago were praised by Darwin [2] as one of the most important organisms in the history of the world. They are widely distributed in many habits, occurring in virtually all soils where the moisture and organic content is adequate, and have tremendous importance for general soil function, and for the growth of agricultural crops [3].

Earthworms such as *Eisenia fetida* are probably the oldest standardised soil model-organisms in ecotoxicology, i.e., used to assess environmental contamination [4], and they are being tested globally [5,6,7,8]. The knowledge developed within these ecotoxicological test on earthworms is vast, not only regarding the standard information required for chemical substances tested under REACH (Registration, Evaluation, Authorisation of Chemicals) frameworks, but also regarding a wide diversity of additional biological endpoints. For these worms, assays can be conducted both using in vitro (cells) and in vivo (whole organisms) systems [7,9], and the endpoints cover information on, e.g., the genome, all omics (transcriptomics, metabolomics, proteomics), the innate immune system, and all of the organism’s life cycle stages (cocoons, juveniles, adults) [10,11]. Hence, there is an excellent understanding of the physiology of these organisms.

The various earthworm species live in a wide diversity of habitats and have had to find solutions—adaptations—for severe environmental challenges. The species *Eisenia fetida* has developed a unique mechanism to deal with intruding (nano)materials, bacteria, and viruses.

In our studies on the potential hazards of nanomaterials [10,12,13], searching for the mechanism of how nanomaterials affect organisms, we have found that nanomaterials become covered by protein coronas when exposed to biological media or in an organism. For *Eisenia fetida*, the dominant protein in the corona is lysenin, which is rapidly induced and highly regulated. Lysenin is likely to have evolved in response to biological invaders, e.g., a virus [14,15]. Hence, the worms treat nanoparticle intruders as it treats biological intruders.

## 2. Earthworm and COVID-19

This led us to wonder if the humble earthworm could have a mechanism that counters infection by enveloped viruses, such as SARS-CoV-2 (Severe Acute Respiratory Syndrome Coronavirus 2, COVID-19), Severe Acute Respiratory Syndrome (SARS), and Middle East Respiratory Syndrome (MERS).

COVID-19 research covers a wide range of specific internal infection (and infection countering) mechanisms, and/or corona viral entry points [16,17,18,19,20,21,22,23]. However, it has long been realized that a weak point of enveloped viruses may be the lipid layer, e.g., of the SARS-CoV-2 envelope [24]. The targeting of viral lipids or host lipids required for viral assembly may represent an effective virucidal approach to develop new anti-viral drugs [25,26,27]. Such lipids, which include sphingolipids, may also interfere with SARS-CoV-2 by binding to ACE-2 (Angiotensin-Converting Enzyme 2) [25,28].

*Eisenia fetida* has a well-known extracellular target defence mechanism involving pore-forming proteins (PFPs) that target lipids [14,15]. PFPs are produced by chloragogen and coelomic cells as soluble proteins that migrate toward the target membrane where they bind with high affinity. These proteins are a group of closely related proteins: lysenin (41 Kda), fetidin (40/45 Kda), and eiseniapore (38 Kda), which respond immediately to “foreign material” invading the worms [10,12,13,14,15,29]. Lysenin and fetidin bind strongly to sphingomyelin (SM, a sphingolipid), destabilize the cell membranes, and cause lysis [30,31]. The process consists of PFPs, e.g., lysenin, forming three (inner)–10 (outer) nm wide pores in the invading “materials/organisms’” membrane [32,33,34]. Sphingomyelin is present in most cell membranes, including the SARS-CoV-2 envelope, in which it may have a higher abundance [35,36,37,38]. In particular, lysenin and fetidin target SM, whereas the eiseniapore is not solely dependent on SM but also binds to galactosylceramide [32,39]. Although the mechanism and structure are well-understood, the exact biological function of lysenin and fetidin is not fully understood, since invading bacteria or fungi rarely have SM in the membrane [40]; however, invading eukaryotes and many enveloped viruses will contain SM. Hence, lysenin (and fetidin) are excellent for defending against enveloped viruses due to their SM specificity and should be further explored.

The virus acts by hijacking the target cells’ metabolic system, altering their lipid metabolism so that it produces the lipid rafts needed by the virus for formation and for replication, which, in some cases, contain substantial enrichment of the sphingolipids [37,38,39,41,42,43,44,45,46]. There are indications that both cellular and viral SM is required for virus infections of Influenza type A virus [35], for HIV [42], for West Nile virus [46], and SARS-CoV [47,48,49]. Moreover, SM depletion in influenza A viruses’ envelopes reduces their ability to infect cells [50], and crude extracts of *E. fetida* tissues can inhibit their cytotoxicity by 69–100% [51]. Hence, worms are unlikely to be infected by SARS-CoV-2 or other envelope (corona) embedded virus, and may be better at handling other viruses, such as Ebola virus and HIV, which require SM and sphingomyelinase activity for infectivity [52].

The earthworm avoids internal damage (self-destruction) by the PFPs (e.g., lysenin) because they are virtually absent of SM [53,54]. Sadly, of course, we humans are not similar to worms and do have SM in our cell membranes, with some areas enriched in SM, e.g., in the myelin layer of nerve axons or exosomes [55]. Hence, to directly inject native *E. fetida* PFPs, e.g., lysenin, into humans will not provide an effective approach to prevent or treat human viral infections.

## 3. Exploration

However, explorations of traditional and new potential applications of *E. fetida* in medicine [1,56] could be extended to include a possible exploitation of earthworms’ virucidal and anti-viral mechanisms to fight infections by SARS-CoV-2 and related corona viruses. For example, by modifying the N-terminal [30,31,34], it is possible to synthesize a modified version of lysenin that is not toxic to human cells (NT-Lysenin) but that still selectively binds to SM clusters (5–6 SM) in membranes [57]. It is also possible to tag lysenin, e.g., with polyhistidine or fluorescence tags [58], and this is often used to identify SM in membranes. Further, it is possible to module the action of the lysenin-induced channels by interactions with multivalent inorganic and organic cations [59].

**Figure 1:**
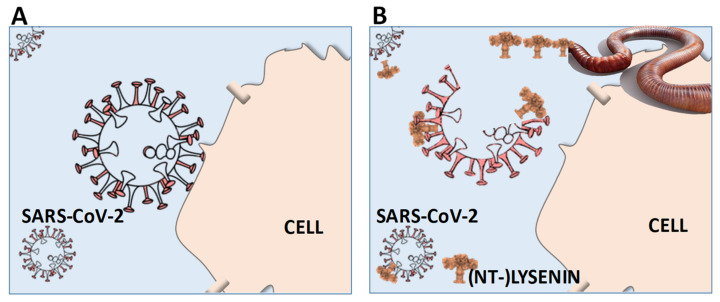
Schematic (hypothetical) outline of the therapeutic potential of lysenin (-derivatives) to prevent or treat human SARS-CoV-2 (COVID-19) infections. (**A**) No action: SARS-CoV-2 enters the organism and attaches to the cell membranes, allowing the RNA injection and infection. (**B**) (NT-)lysenin administration: SARS-CoV-2 enters the organism and encounters lysenin, which binds to sphingomyelin in SARS-CoV-2 envelope (corona), forming pores and disrupting it, hence no cell membrane attachment and, thus, no infection. (NT-)LYSENIN: Non-Toxic-Lysenin: representation of a modified lysenin derivative.

As mentioned above, the virus membrane differs from that of the host and the virus highjacks the cells’ metabolic system to alter the lipid metabolism so that it produces the lipid rafts needed by the virus for replication [37,38,44,60]. Thus, it may be possible to target this binding process therapeutically (Figure 1) if the SM distribution differs sufficiently between human and viral membranes, as shown for HIV and West Nile virus where the SM level is (e.g., 2–3 times) higher than the target cell [46,61,62,63]. Further, for SARS-CoV (and HIV), the virus binding is both dependent on the lipid rafts (sphingomyelin and cholesterol) and the proteins (e.g., ACE2) in the cell membrane, hence NT-lysenin may be used to study whether there is a reorganisation of SM in the cell surface of the target cell and of the virus [47,48,49,64]. It may be noted that SM plays a particular role in determining the cholesterol accessibility (due to high SM affinity to cholesterol [65]) and that higher cholesterol availability in membrane coincides with SARS-CoV-2 entry [66]. Other alternatives, to ensure that lysenin-derivatives specifically bind to viral membranes, may also emerge if researchers explore their therapeutic potential, e.g., developing SARS-CoV-2-related molecular traps [18]. Since it is possible to tag lysenin, lysenin can also deliver drugs attached to the N-terminal [58,67]. In addition, the delivery of lysenin can be targeted to specific tissues if they are carried with nanocarriers (inside or outside), e.g., gold nanoparticles, lipid- or polyethylene glycol (PEG)-carriers, as used for vaccines [68,69]. The delivery carrier should obviously be chosen for this specific purpose, and it should be considered that lysenin may interact with the nanocarrier itself, e.g., if a sphingomyelin-based liposome is used [70,71]. Hence, various model carriers should be tested, e.g., some of the carriers that are already approved for vaccines. Further, earthworms contain a number of other interesting anti-infection compounds, e.g., lumbricins [72]. Obviously, similar considerations and investigations should be given to the two other earthworm PFPs, fetidin and eiseniapore, perhaps as a combined approach. To support the approach, it is well-known that pore-forming proteins have important biological functions and that these can be therapeutically effective, e.g., against cancer, inflammatory diseases, and immunomodulation [68,73,74,75]. Given this, it seems prudent to take an extra look at this humble earthworm to help us develop new treatments, also in the light of the mutation in the spike protein.

## 4. Conclusions

More studies should be conducted to explore the kinetic processes of PFPs formation, topological accumulation, and dynamic alterations of the sphingomyelin pool in human airway epithelial cells. Careful consideration must also be given to the potential risk of toxicity elicited by lysenin for in vivo usage. It is worth noticing that there is a good understanding of lysenin’s binding to sphingomyelin clusters in cell membranes, cells producing these proteins are easily extracted from the worms, and that the species (*E. fetida*) is present in many laboratories across the world, since they have been OECD standard test species for environmental toxicology for more than 25 years. Hence, organizations such as the WHO (World Health Organization) can quickly mobilize many scientists to explore the possibility of using the pore-forming proteins to target viruses and verify the efficiency of the presented hypothesis.

## Data Availability

All data are available in the main text.
